# Polymeric Micelles for Apoptosis-Targeted Optical Imaging of Cancer and Intraoperative Surgical Guidance

**DOI:** 10.1371/journal.pone.0089968

**Published:** 2014-02-26

**Authors:** Hyunah Cho, Clifford S. Cho, Guilherme L. Indig, Afsaneh Lavasanifar, Mohammad Reza Vakili, Glen S. Kwon

**Affiliations:** 1 Pharmaceutical Sciences Division, School of Pharmacy, University of Wisconsin, Madison, Wisconsin, United States of America; 2 Section of Surgical Oncology, Department of Surgery, University of Wisconsin, Madison, Wisconsin, United States of America; 3 Department of Chemistry and Biochemistry, University of Wisconsin, Milwaukee, Wisconsin, Unites States of America; 4 Faculty of Pharmacy and Pharmaceutical Sciences, University of Alberta, Edmonton, Alberta, Canada; University of Utah, United States of America

## Abstract

In a two-step strategy, an intraperitoneal (IP) injection of poly(ethylene glycol)-*block*-poly(ε-caprolactone) (PEG-*b*-PCL) micelles containing paclitaxel (PTX), cyclopamine (CYP), and gossypol (GSP) at 30, 30, and 30 mg/kg, respectively, debulked tumor tissues by 1.3-fold, based on loss of bioluminescence with <10% body weight change, and induced apoptosis in peritoneal tumors when used as neoadjuvant chemotherapy (NACT) in an ES-2-luc-bearing xenograft model for ovarian cancer. In a second step, a single intravenous (IV) injection of apoptosis-targeting GFNFRLKAGAKIRFGS-PEG-*b*-PCL micelles containing a near-infrared (NIR) fluorescence probe, DiR (1,1′-dioctadecyltetramethyl indotricarbocyanine iodide), resulted in increased peritoneal DiR accumulation in apoptosis-induced ES-2-luc tumor tissues (*ex vivo*) by 1.5-fold compared with DiR molecules delivered by methoxy PEG-*b*-PCL micelles (non-targeted) at 48 h after IV injection in a second step. As a result, a tandem of PEG-*b*-PCL micelles enabled high-resolution detection of *ca*. 1 mm diameter tumors, resulting in resection of approximately 90% of tumors, and a low peritoneal cancer index (PCI) of *ca*. 7. Thus, a tandem of PEG-*b*-PCL micelles used for NCAT and NIR fluorescence imaging of therapy-induced apoptosis for intraoperative surgical guidance may be a promising treatment strategy for metastatic ovarian cancer.

## Introduction

Ovarian cancer is portrayed as the disease that whispers due to the indolent symptoms at early stages [1]. Unfortunately, there are no effective screening tests: general screening of serum cancer antigen 125 (CA 125) or transvaginal sonography does not permit early detection of ovarian cancer. In part due to the difficulties in diagnosis, ovarian cancer is the most lethal gynecologic malignancy [2]. The conventional treatment strategy for ovarian cancer involves a combined approach utilizing aggressive cytoreductive surgery and intravenous (IV) chemotherapy (platinum and taxane analogues) [3].

In the last decade, a potential benefit of chemotherapy given through the intraperitoneal (IP) route for ovarian cancer has been seen in several clinical trials, and it has been highlighted that IP chemotherapy can give high response rates within the abdomen due to the peritoneal plasma barrier confining exposure of chemotherapy to peritoneal surfaces, resulting in higher drug concentration in peritoneal cavity [3].

Surgery is critical for patients with colorectal and ovarian cancers that have spread widely to the peritoneal cavity. There are three types of surgical debulking that have been attempted to treat ovarian cancer patients: (1) primary debulking surgery (initial surgery), which has largely been the standard approach; (2) interval debulking surgery after neoadjuvant chemotherapy (NACT), reserved for patients who are medically unfit for immediate operation or whose extensive metastases cannot be initially resected; and (3) secondary debulking surgery (additional surgery), for patients who develop recurrent chemoresistant ovarian cancer [1]. Although the optimal surgical approach remains controversial, it is very clear that improved intraoperative cancer imaging systems will yield significant benefit for successful surgical debulking of ovarian cancer. Although radiological approaches such as computed tomography (CT) and magnetic resonance imaging (MRI) have been of great help in characterizing malignancies within peritoneal cavity, they are not useful for intraoperative assessment. In contrast, fluorescence imaging has been shown to be successful in preclinical and clinical trials as an optical technique offering real-time images of surgical targets (peritoneal carcinomatosis and breast cancer) with adequate imaging resolution and high intraoperative sensitivity [4]–[8]. Coll and colleagues reported that intraoperative near-infrared (NIR) fluorescence image-guided surgery using a tumor-targeting peptide, RAFT-c(RGDfK)_4_-Alex Fluor 700 (IV route), in a TSA-pGL3-bearing mouse model of peritoneal adenocarcinoma could improve the quality of surgical debulking by doubling the number of detected tumor nodules and shortening operation time [5]. Ntziachristos and colleagues successfully conducted the first human trial of intraoperative tumor-specific fluorescence imaging in staging and debulking surgery for ovarian cancer using IV folate-FITC, and proved that the number of tumors detected by surgeons under the guidance of tumor-specific fluorescence images increased by 5.3-fold (34 *vs.* 7) compared with white light visual observation alone [7]. Frangioni and co-workers also demonstrated the utility of the FLARE (Fluorescence-Assisted Resection and Exploration) device for image-guided oncologic surgery in the first human clinical trial of breast cancer sentinel lymph node (SLN) mapping following intratumoral/subcutaneous injection of 1∶1 mixture of indocyanine green and human serum albumin (ICG:HAS) [6]. In this trial, SLNs identified by lymphoscintigraphy and NIR fluorescence imaging were identical in 4 of 6 breast cancer patients.

Application of nanomaterials as optical fluorescence imaging agents using quantum dots, gold nanoparticles, and fluorescence probe-containing or -conjugated nanoparticles has drawn attention for diagnostic purposes in pre-clinical studies [9]–[13]. Polymeric micelles belong to a major class of nanomaterials that have entered several clinical trials for drug delivery, *eg*. SP-1049C (doxorubicin, phase II), Genexol-PM (paclitaxel, phase II), and NC-6004 (cisplatin, phase I) [14]. Polymeric micelles offer several advantages not only as drug carriers but also as optical imaging agents in oncology: small sized-particles with narrow size distribution, structural stability, high water solubility, low toxic side effect over conventional surfactants (*eg*. Cremophor EL), preferential accumulation at solid tumors through enhanced permeability and retention (EPR) effect, evading renal filtration, and multifunctionality by surface decoration.

It has been discussed that apoptosis-targeted drug delivery and cancer imaging (specially for imaging tumor responsiveness to chemotherapy) [15], [16] could be superior than cancer-associated antigen- or protein-targeting strategy in a broad range of malignancies, because substantial heterogeneity in cancer cell populations does not guarantee the exclusive presence of antigen and protein biomarkers in target tissues [17]. Surgical oncologists could take advantage of apoptosis-targeted tumor imaging (independent of cell type and cell death-inducing triggers) after NACT with greater precision and accuracy [18]. Thus, surgical tumor debulking using intraoperative visual guidance with real-time NIR fluorescence images could result in improved surgical accuracy and outcome. One of the most prominent characteristics of programmed cell death, apoptosis, is the externalization of phosphatidylserine (PS) which normally resides predominantly in the inner leaflet of the plasma membrane [18].

In our previous work, we reported that poly(ethylene glycol)-*block*-poly(ε-caprolactone) (PEG-*b*-PCL) micelles containing DiR (1,1′-dioctadecyltetramethyl indotricarbocyanine iodide) could passively accumulate in LS180 human solid colon tumor tissues by EPR effect and provide noninvasive delineation of LS180 tumor tissues with a tumor-to-muscle ratio of 30–43 from collected tissues [19]. We also observed enhanced DiR accumulation in “primed” LS180 tumor tissues after an IV injection of multi-drug containing poly(ethylene glycol)-*block*-poly(_D,L_-lactic acid) (PEG-*b*-PLA) micelles, suggesting the availability of a tandem of polymeric micelles that could possibly enable improved tumor delineation for use in surgical oncology [20]. More recently, PEG-*b*-PCL micelles with paclitaxel (PTX), cyclopamine (CYP), and gossypol (GSP) at 30, 30, and 30 mg/kg, respectively (q7d×3) delivered through IP, prevented the metastatic spread of ovarian cancer and extensive ascites formation, resulting in prolonged survival in peritoneally metastatic ES-2-luc (undifferentiated adenocarcinoma, aggressive subtype) and SKOV-3-luc (serous adenocarcinoma, moderate-grade subtype) murine xenograft models of ovarian cancer [21].

**Figure 1:**
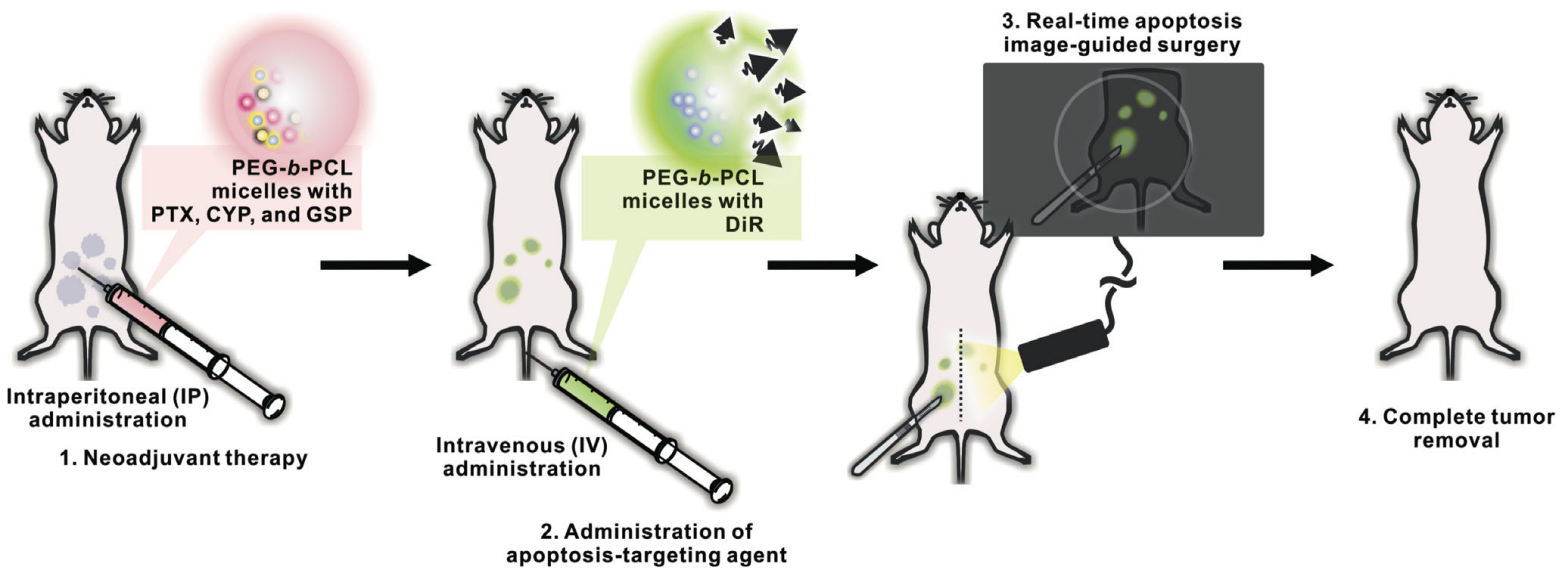
Schematic illustration of two-step strategy for neoadjuvant therapy, apoptosis-targeted optical imaging and intraoperative surgical guidance, enabled by a tandem of PEG-*b*-PCL micelles.

We propose a novel two-step strategy for NACT, apoptosis-targeted optical imaging and intraoperative surgical guidance (Figure 1). In step one, PEG-*b*-PCL micelles containing PTX, CYP, and GSP are used for IP NACT and apoptosis induction in tumor tissues. In a second step, apoptosis-targeting PEG-*b*-PCL micelles containing DiR may actively accumulate in apoptotic tumor tissues, permitting optical fluorescence imaging of apoptosis in a real-time manner (Figure 1). DiR molecules, NIR fluorescence probes emitting light in the NIR wavelength window, could be useful as an optical fluorescence imaging agent, avoiding strong autofluorescence from skin and blood and allowing detectable signals to be measured through several millimeters of tissues [22]. In this work, we show that this two-step strategy enhanced tumor delineation in NIR fluorescence optical imaging and provided useful guidance for interval debulking surgery in an IP ES-2-luc-bearing xenograft model of ovarian cancer, coupling two applications of polymeric micelles in drug delivery and optical imaging for surgical oncological therapy of ovarian cancer.

## Materials and Methods

### Preparation of PEG-*b*-PCL Micelles Carrying PTX, CYP, and GSP

Drug-loaded PEG-*b*-PCL micelles were prepared by a solvent evaporation method as previous described [21]. Briefly, 4.0 mg of PTX, CYP, and GSP each and 120 mg of PEG-*b*-PCL (M_n_ of PEG = 5,000 g/mol, M_n_ of PCL = 10,000 g/mol, and M_w_/M_n_ = 1.26) (Advanced Polymer Materials Inc., Montreal, Canada) were completely dissolved in 1.0 mL of acetone followed by a rapid addition of 1.0 mL of pre-warmed 0.9% saline at 60°C with vigorous mixing. Acetone was evaporated under reduced pressure at 60°C. The aqueous micelle solution was centrifuged for 5 min at 10,000 ×*g* and passed through a 0.2 µm regenerated cellulose (RC) sterile syringe filter (Corning, Tewksbury, MA) to remove insoluble drugs. The content of PTX, CYP, and GSP in PEG-*b*-PCL micelles was quantified by reverse phase-HPLC (RP-HPLC) system using a Shimadzu Prominence HPLC system (Himadzu, Japan) as previously described [21]. The separation of PTX, CYP, and GSP was done in an isocratic mode with mobile phase of 55% acetonitrile, 45% distilled water, and 0.1% trifluoroacetic acid. PTX, CYP, and GSP were monitored at 227, 204, and 373 nm, respectively, and eluted at 2.7 min, 1.9 min, and 10.6 min, respectively.

### Preparations of Apoptosis-targeting PEG-*b*-PCL and Methoxy-PEG-*b*-PCL Micelles Carrying DiR

**Figure 2:**
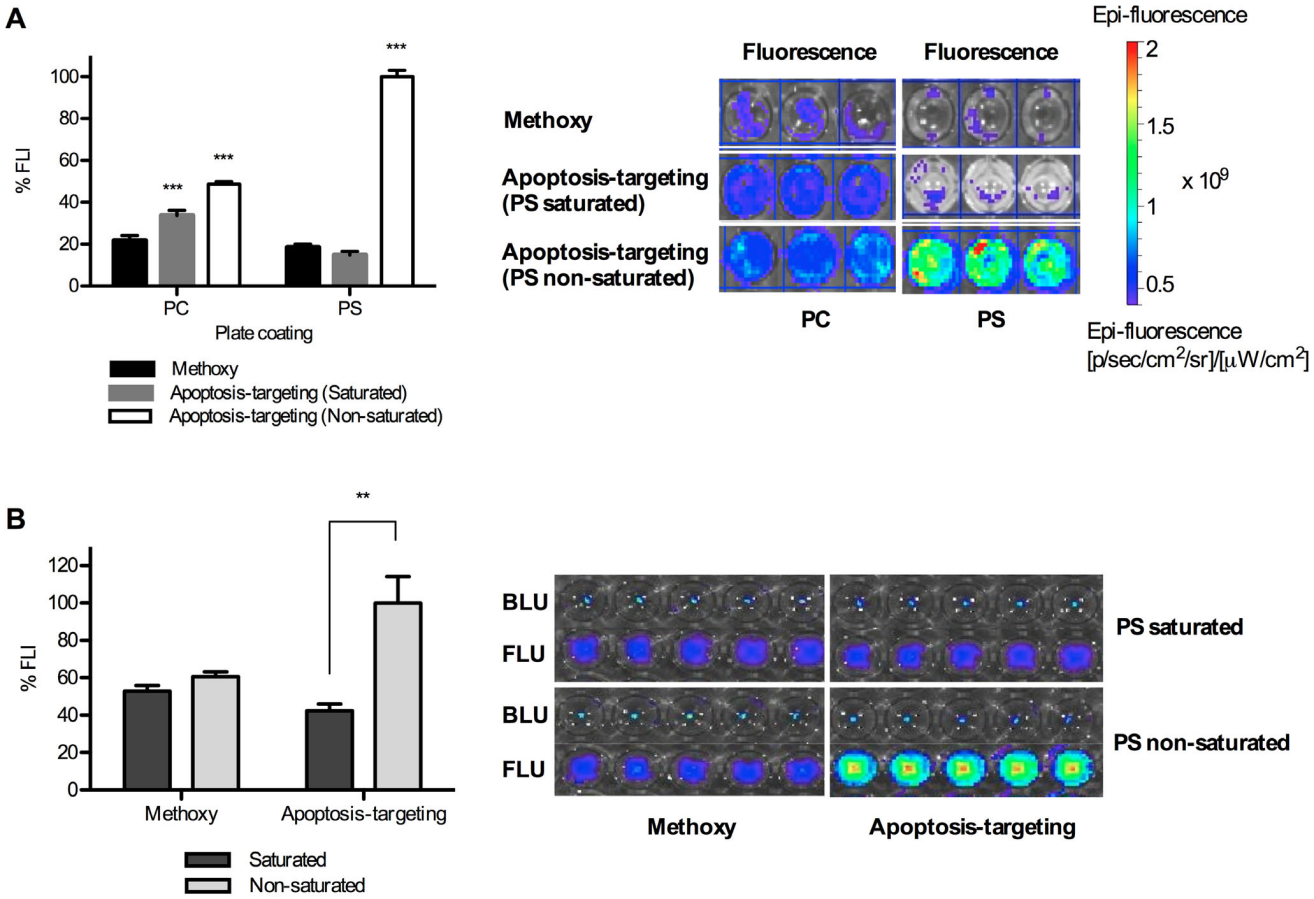
Assessment of competitive PS- or apoptosis-binding of apoptosis-targeting PEG-*b*-PCL micelles carrying DiR by PS saturation with free peptides *in vitro*. Results are presented as %FLI DiR molecules bound on plates and %FLI from DiR molecules bound on ES-2-luc ovarian tumor spheroids. BLI from ES-2-luc cells and FLI from DiR molecules were quantified using Xenogen IVIS 200 Series. (A) Competitive binding test of apoptosis-targeting PEG-*b*-PCL micelles carrying DiR (1.0 µM peptide and 500 nM DiR) to PC- or PS-coated 96-well plates (total 200 µM of phospholipid). (B) Competitive binding test of apoptosis-targeting PEG-*b*-PCL micelles carrying DiR to apoptosis-induced ES-2-luc ovarian tumor spheroids (** <0.01, *** <0.001).

Preparation of apoptosis-targeting PEG-*b*-PCL (GFNFRLKAGAKIRFGS-PEG-*b*-PCL) micelles carrying DiR was started with the conversion of acetal groups on the surface of PEG-*b*-PCL micelles to aldehyde groups (Figure S1). Acetal-PEG-*b*-PCL (M_n_ of PEG = 5,000 g/mol, M_n_ of PCL = 5,000 g/mol, and M_w_/M_n_ = 1.13) was kindly provided by Dr. Afsaneh Lavasanifar, University of Alberta (Edmonton, Canada). The conversion method was slightly modification from the literature [23]. Acetal-PEG-*b*-PCL copolymer was dissolved in acetone at a concentration of 20 mg/mL. Distilled water was then rapidly added to the polymer solution with vigorous stirring at room temperature followed by evaporation of acetone under reduced pressure at room temperature. The micelle solution was centrifuged for 5 min at 10,000 ×*g* and passed through a 0.2 µm RC sterile syringe filter. Conversion of acetal groups on acetal-PEG-*b*-PCL micelles was carried out at pH 2.0 by adding 0.5 N of HCl. After 4 h of moderate stirring at room temperature, the reaction was neutralized with 0.5 N of NaOH to stop the reaction. The neutralized micelle solution was then dialyzed against water with dialysis membrane (MWCO 6,000 g/mol) to remove the salt overnight and lyophilized for 48 h for the future use. Lyophilized sample was dissolved in CDCl_3_ (6 mg/mL) to estimate the conversion rate from the acetal to aldehyde group on PEG-*b*-PCL by ^1^H NMR. Free peptide, GFNFRLKAGAKIRFGS (UW Biotechnology Center, Madison, WI) at Mw = 1,769 g/mol (Figure 2A), was dissolved in HEPES buffer (10 mM, pH 6.4) and mixed with the lyophilized aldehyde-PEG-*b*-PCL micelles to obtain 4 mg/mL of polymer and 0.35 mg/mL of peptide concentration at 2∶1 molar ratio (aldehyde-PEG-*b*-PCL:GFNFRLKAGAKIRFGS). After 2 h of moderate stirring, NaBH_3_CN (10 equiv) was added to the mixture to reduce Schiff base. After 4 days, micelle solution was again dialyzed against water with dialysis membrane (MWCO 6,000 g/mol) overnight and then GFNFRLKAGAKIRFGS-PEG-*b*-PCL micelle solution was lyophilized for 48 h. The conjugation efficiency of peptide on aldehyde-PEG-*b*-PCL was determined by RP-HPLC analysis. Briefly, samples (10 µL) were injected into a Zorbax 300SB-C18 column (4.6×15 mm, 3.5 µm, Agilent) kept at 40**°**C and the flow rate was 0.8 mL/min. Gradient elution was performed with the mobile phase of 0.1% trifluoroacetic acid in distilled water and 0.1% trifluoroacetic acid in 90/10 (v/v) acetonitrile/distilled water. The mobile phase was programed as follows: 0 min 85% solvent A and 15% solvent B; 35 min, 50% solvent A and 50% solvent B. Free peptide was monitored at 215 nm and eluted at 16 min. The amount of peptide conjugated on PEG-*b*-PCL micelles was calculated by subtracting the amount of free peptide from the amount of peptide initially added to reaction. In parallel, lyophilized peptide conjugated on PEG-*b*-PCL was dissolved in DMSO-*d*_6_ (6 mg/mL) to calculate conjugation rate of peptide on PEG-*b*-PCL by ^1^H NMR at 80°C.

Methoxy-PEG-*b*-PCL and apoptosis-targeting PEG-*b*-PCL micelles carrying DiR were prepared by a solvent evaporation method: 4.0 mg of polymers and 0.1 mg of DiR (Invitrogen, Carlsbad, CA) were dissolved in 1.0 mL of acetone, followed by a rapid addition of PBS (10 mM, pH 7.4) with vigorous mixing. Acetone was evaporated under reduced pressure at room temperature. The aqueous micelle solution containing DiR was centrifuged for 5 min at 10,000 ×*g* and passed through a 0.2 µm RC sterile syringe filter to remove unincorporated DiR. The content of DiR in micelles was quantified by RP-HPLC as previously described [20]. The elution of DiR was done in a gradient mode with mobile phase of 70% distilled water and 0.07% trifluoroacetic acid as a solvent A and 30% acetonitrile and 0.03% trifluoroacetic acid as a solvent B. DiR was monitored at 745 nm and eluted at 16 min.

### Physical Characterization of Methoxy-PEG-*b*-PCL and Apoptosis-targeting PEG-*b*-PCL Micelles Carrying DiR

Z-average diameters of methoxy-PEG-*b*-PCL micelles and apoptosis-targeting PEG-*b*-PCL micelles carrying DiR were determined by dynamic light scattering (DLS) measurements using Zetasizer Nano-ZS (Malvern Instruments, United Kingdom) at 25°C with a detection angle of 173° and a He-Ne ion laser (4 mW, λ  =  633 nm) for the incident beam. Autocorrelation functions were created based on cumulant analysis, calculating the hydrodynamic diameter of micelles from the Stokes-Einstein equation and the polydispersity index (PDI). Prior to measurements, micelle solutions were diluted with PBS (10 mM, pH 7.4) to give a polymer concentration at ∼ 0.4 mg/mL that represents the polymer concentration above the critical micelle concentration. DiR loading efficiency was shown as % weight DiR/weight polymer. The *in vitro* DiR release kinetics of methoxy-PEG-*b*-PCL and apoptosis-targeting PEG-*b*-PCL micelles carrying DiR was studied to estimate the time for 50% drug release (t_1/2_) based on a one-phase decay model using GraphPad Prism version 5.00 for Mac OS X (La Jolla, CA). DiR-loaded micelles, representing 100 µg/mL of DiR, were added into dialysis cassettes (MWCO 20,000 g/mol), and cassettes were placed in 2.0 L of PBS (10 mM, pH 7.4) at 37°C with moderate stirring. Samples (20 μΛ were withdrawn from cassettes at various time points, 0, 0.5, 1, 2, 3, 8, 12, and 24 h and after each sampling, cassettes were replenished with 20 μΛ of fresh PBS (10 mM, pH 7.4). The content of DiR in micelles left in cassettes was analyzed by RP-HPLC as described above.

### Assessment of PS-selective Binding of Apoptosis-targeting PEG-*b*-PCL Micelles Carrying DiR

Binding of apoptosis-targeting PEG-*b*-PCL micelles carrying DiR to PS was assessed using phospholipid-coated well plates [24] and 3-D tumor spheroids formed from luciferase-expressing ES-2-luc cells. PC or PS solubilized in ethanol was immobilized on clear-bottom 96-well plates at a concentration of about 200 µM in each well, and ethanol was evaporated at room temperature overnight. Some of PC or PS-coated wells were incubated with 1.0 µM of free peptide for 3 h to saturate PS on phospholipid-coated wells. Apoptosis-targeting PEG-*b*-PCL micelles carrying DiR, representing 1.0 µM of peptide and 500 nM of DiR, were added to wells and incubated for 1 h at room temperature in dark. Each well was washed with PBS (10 mM, pH 7.4) and apoptosis-targeting PEG-*b*-PCL micelles carrying DiR bound on wells was detected by Xenogen IVIS 200 Series (Caliper Life Sciences, Hopkinton, MA). ES-2-luc 3-D tumor spheroids were generated by plating 5,000 ES-2-luc cells/well in agarose-coated 96-well plates and incubated for 4 days. ES-2 human ovarian cancer cells were stably transfected with luciferase-expressing plasmid pGL4.51 containing the neomycin-resistance gene (Promega, Madison, WI) using Lipofectamine 2000 (Invitrogen, Carlsbad, CA) as previously described [21]. ES-2-luc cells were cultured in McCoy’s 5a medium supplemented with 1% L-glutamine, 10% fetal bovine serum, 1% penicillin/streptomycin, and 750 µg/mL G418 antibiotics and maintained at 37°C under an atmosphere of 5% CO_2_ in a humidified incubator. PEG-*b*-PCL micelles containing PTX, CYP, and GSP (3.3, 3.3, and 3.3 nM, respectively) were added to ES-2-luc tumor spheroids and incubated for 3 days. Some of treated ES-2-luc spheroids were incubated 3 h with 200 nM of free peptide to saturate PS exposed on tumor spheroids. Apoptosis-targeting PEG-*b*-PCL micelles carrying DiR were then added to ES-2-luc tumor spheroids at a final concentrations of 200 nM peptide and 100 nM DiR and incubated for 30 min at 37°C in 5% CO_2_ humidified incubator. As a control, bioluminescence intensity of ES-2-luc cells was also monitored to assure that ES-2-luc tumor spheroids were consistently formed in each well, using Xenogen IVIS 200 Series.

### Intraperitoneal Human Ovarian Cancer Xenograft and Micelle Treatments

Female 6–8 week-old athymic nude Foxnl^nu^ mice were purchased from Harlan Laboratories (Madison, WI). All animal experiments were carried out in strict accordance with the recommendations in the Guide for the Care and Use of Laboratory Animals of the National Institutes of Health (NIH). The ethical and humane use of animals was advised by the All Campus Animal Planning and Advisory Committee (ACAPAC) and the protocol was approved by the Institutional Animal Care and Use Committee (IACUC) at the University of Wisconsin-Madison (Permit number: M02472). General anesthesia was induced with 1.5% isoflurane/oxygen and maintained with 1% isoflurane/oxygen. ES-2-luc cells were trypsinized, collected from sub-confluent cultures, and 1×10^6^ cells/animal of ES-2-luc cells were injected into the peritoneal cavity of anesthetized mice. IP injection of PEG-*b*-PCL micelles carrying 30, 30, and 30 mg/kg of PTX, CYP, and GSP was performed 7 days post IP inoculation of ES-2-luc cells after observation of bioluminescence signal in whole-body images of animals by Xenogen IVIS 200 Series. One day after IP injection of PEG-*b*-PCL micelles containing PTX, CYP, and GSP, methoxy or apoptosis-targeting PEG-*b*-PCL micelles carrying 250 µg/kg of DiR was injected through the tail vein of anesthetized animals. Body weights of animals were measured by a portable scale, and general appearance and mortality of animals was carefully monitored during all sets of animal experiments. All efforts were made to minimize suffering of animals. Animals were euthanized by medical grade carbon dioxide with the flow rate of 10–30% of the euthanasia chamber volume per minute.

### TUNEL Assay

ES-2-luc-bearing xenograft was dosed through IP route with PEG-*b*-PCL micelles carrying PTX, CYP, and GSP on day 7 after ES-2-luc cells were inoculated in the peritoneal cavity. Afterwards, animals (*n* = 4) were sacrificed at 0, 12, 24, 48, and 72 h post micelle treatment. Tumor, spleen, kidney, and liver tissues were dissected. DNA fragmentation induced by apoptosis was detected in tissues by TdT-mediated dUTP Nick-End Labeling (TUNEL) method using DeadEnd Fluorometric TUNEL assay kit (Promega, Madison, WI). Tissues were frozen sectioned into 10 µm slices, permeabilized by proteinase K, and fixed with 4% formaldehyde. The reaction mixture consisting of TdT and fluorescein-labeled dUTP was added to fixed section of tissues and incubated for 1 h at 37°C in a humidified chamber in dark. Fluorescein-labeled DNA fragments and nuclei of cells couterstained by DAPI were visualized at 520 nm and 460 nm, respectively, using a confocal microscope (Olympus FV1000 FLUOVIEW, Minneapolis, MN). Apoptotic cells and nuclei of cells were shown in green and blue, respectively.

### Bioluminescence and Fluorescence Imagings

Xenogen IVIS 200 Series was used to image both bioluminescence and fluorescence from objects *in vitro*, *in vivo*, and *ex vivo*. Xenogen IVIS 200 Series was equipped with a 150 W quartz halogen lamp and a 1 mW power scanning laser. Images were screen-displayed with the spatial resolution of >60 µm/pixel. Bioluminescence images were acquired by a charged couple device (CCD) camera with the following parameters: exposure time = 1 s, binning = medium, and f/stop = 2. *In vitro*, D-luciferin (Caliper Life Science, Hopinton, MA) at 10 µg/well was added to ES-2-luc tumor spheroids 5 min prior to bioluminescence imaging, and *in vivo*, D-luciferin at 113 mg/kg was injected IP into ES-2-luc-bearing xenograft model 5 min prior to whole-body bioluminescence imaging. The dynamic of ES-2-luc tumor growth was collected and shown as color-coded images using Live Imaging software for quantitative analysis and BLI of ES-2-luc tumors was scaled by total counts.

Fluorescence images were also acquired by a charged couple device (CCD) camera with the following parameters: exposure time = 1 s, binning = medium, and f/stop = 2. A filter setting for DiR detection was fixed at 745 nm for excitation and at 800 nm for emission. All color-coded images were collected using Live Imaging software for image acquisition and analysis. FLI of DiR was demonstrated by average radiant efficiency, total photons per second per square centimeter per steradian in the irradiance range (microwatts per square centimeter): [ps^−1^cm^−2^sr^−1^]/[µW cm^−2^]. The fluorescence of DiR in tumor tissues was determined from the average radiant efficiency at ROI drawn around tumor tissues preset by bioluminescence imaging of an identical animal.

Whole-body bioluminescence and fluorescence images were recorded at 6, 12, 24, and 48 h post an IV injection of PEG-*b*-PCL micelles carrying DiR. Mice were placed in abdominal positions to obtain whole-body color-coded bioluminescence and fluorescence images. All equipment settings for bioluminescence and fluorescence imagings were identical in the time course experiment.

### Real-time Fluorescence Imaging Acquisition in Animals

Fluobeam 800 is a 2-D NIR fluorescence imaging system composed of CCD camera and an integrated NIR light source (100 mW) with an excitation wavelength at 780 nm and an emission wavelength at >820 nm. This system provided 7.5×10 cm of homogeneous lightened field and the portable hand-held system composed of camera and laser was located approximately 20 cm above the object. Fluorescence images were screen-displayed with the spatial resolution of 110 µm/pixel and recorded as either black-and-white static images (9 images/sec) or real-time videos (25 images/sec).

### Surgical Procedure

Animals received an IP injection of D-Luciferin (113 mg/kg) 5 min prior to the whole-body bioluminescence imaging on day 9 after ES-2-luc cell inoculation (24 h post IV injection of PEG-*b*-PCL micelles carrying DiR). After whole-body bioluminescence imaging was carried out, animals were sacrificed immediately. All sacrificed animals underwent a midline laparotomy and bioluminescence whole-body images of incised animals were obtained again. All surgical procedures were performed on sacrificed animals and by a surgical oncologist experienced in murine surgery. In one set of experiments (*n* = 4), for comparison, the traditional surgical tumor resection was performed under the normal white-light. When surgical tumor resection was deemed satisfactory, dissected tumor-like tissues and carcass were scanned by Xenogen IVIS 200 Series to obtain bioluminescence images. In another group of animals (*n* = 4), NIR imaging system, the Fluobeam 800 NIR imaging system was turned on and the surgical tumor resection was assisted by real-time fluorescence images displayed on the screen. When tumor-like FLU^+^ tissues were excised as completely as possible, dissected tumor-like tissues and the carcass were scanned using the Xenogen IVIS 200 Series to obtain bioluminescence and fluorescence images. The duration of surgery was recorded from the time of incision to completion of tumor debulking. Surgical accuracy was assessed using two calculations: (1) % sum of BLU^+^ tumor-like tissues to sum of total tumor-like tissues excised throughout the surgical procedure, and (2) total counts of BLI of [ROI in mid-line incised animal minus ROI in dissected animal] over those of ROI in mid-line incised animal. Surgical accuracy was also measured by calculating a post-resection peritoneal cancer index (PCI) as described by Sugarbaker [3]. The PCI relies on the distribution and size of lesions in the abdomen of animal. In this study, PCI was adapted to tumor sizes in mice with the following scores: The abdomen was divided into 13 regions and the lesion size (LS) was scored (0 to 3) in each region as follows: no visual tumors (LS = 0), >0 to 0.5 mm tumor (LS = 1), 0.6 to 2.0 mm tumor (LS = 2), and >2.0 mm tumor (LS = 3). Total LS score per animal was summated as a numerical score which can be ranged from 0 (no tumors observed) to 39 (13 areas×3).

### Statistical Analysis

Statistical analysis was done using one-way ANOVA at 5% significance level combined with Tukey’s multiple comparison tests by GraphPad Prism ver 5.00 for Mac OS X (La Jolla, CA).

## Results

### Characterization of Apoptosis-targeting PEG-*b*-PCL Micelles Carrying DiR

Table 1 presents sizes and loading efficiencies (% weight DiR/weight polymer) of apoptosis-targeting PEG-*b*-PCL (GFNFRLKAGAKIRFGS-PEG-*b*-PCL) and methoxy-PEG-*b*-PCL micelles achieved after forming DiR-incorporated micelles by a solvent evaporation technique. Apoptosis-targeting PEG-*b*-PCL micelles had a z-average diameter of 83±2 nm (polydispersity index, PDI 0.1) and methoxy-PEG-*b*-PCL micelles had a z-average diameter of 45±2 nm (PDI 0.1). The loading efficiency of DiR for both micelles was approximately 2%. Peptide (GFNFRLKAGAKIRFGS) was conjugated onto PEG-*b*-PCL micelles with the molar conjugation ratio at 30% *via* a Schiff base reaction, based on results from both HPLC (subtractive quantification of non-conjugated peptide from peptide initially added for reaction) and ^1^H NMR (quantification of conjugated peptide) analyses (data not shown). In ^1^H NMR analysis, the relative intensity ratio of the peak of benzyl protons of phenylalanine at δ = 7.2 ppm in peptide to the methylene proton peak of PEG protons at δ = 3.7 ppm determined the level of peptide on PEG-*b*-PCL. The data for peptide quantification from HPLC and ^1^H NMR provided comparable values. No significant difference in t_1/2_ value of DiR for both micelles was observed (t_1/2_ = *ca*. 2 h) with a similar pattern of *in vitro* DiR release.

**Table 1:** Physicochemical properties of PEG-*b*-PCL micelles carrying DiR.

Micelles	Size (nm)	PDI	Peptideconjugation (%)	DiR loadingefficiency (%)	Half-life (t_1/2_) ofDiR release (h)
**Apoptosis-targeting** **PEG-** ***b*** **-PCL micelles**	83.4±1.8	0.1±0.0	30	2.4±0.1	2.2
**Methoxy-PEG-** ***b*** **-PCL** **micelles**	45.1±2.0	0.1±0.0	N/A	2.1±0.2	2.0

### *In vitro* Binding Studies

Binding of apoptosis-targeting PEG-*b*-PCL micelles carrying DiR to PS was validated in both phospholipid PC or PS-coated 96-well plates and apoptosis-induced ES-2-luc tumor spheroids pretreated with PEG-*b*-PCL micelles with PTX, CYP, and GSP as shown in Figure 2. Apoptosis-targeting peptide (GFNFRLKAGAKIRFGS) has the consensus sequence motif, FXFXLKXXXKXR, representing a basic structural motif for the specific interaction with PS [25]. Considering fluorescence intensity (FLI) of DiR delivered by apoptosis-targeting PEG-*b*-PCL micelles to PS-coated wells to be 100%, apoptosis-targeting PEG-*b*-PCL micelles carrying DiR showed lower adsorption onto phosphatidylcholine (PC)-coated wells (49±1%), showing a 2-fold weaker FLI on PC-coated wells from DiR (Figure 2A). In a competitive binding test, free peptide added prior to the binding study substantially blocked the binding of apoptosis-targeting PEG-*b*-PCL micelles carrying DiR to PS and resulted in a 5-fold decreased FLI of DiR in PS-coated plates (15±2%). Binding of methoxy-PEG-*b*-PCL micelles carrying DiR to both PS- and PC-coated wells was equivalent *ca*. 19%, and PS saturation did not significantly affect the binding affinity of methoxy-PEG-*b*-PCL micelles carrying DiR to both PS- and PC-coated wells.

A similar pattern of binding was observed in ES-2-luc tumor spheroids pretreated with PEG-*b*-PCL micelles carrying PTX, CYP, and GSP at 3.3, 3.3, and 3.3 nM, respectively (spheroids maintained their spherical integrity after treatment) (Figure 2B). Assuming PS was externalized by apoptosis induction after treating with PEG-*b*-PCL micelles carrying PTX, CYP, and GSP in equally-sized ES-2-luc tumor spheroids, DiR molecules delivered by apoptosis-targeting PEG-*b*-PCL micelles were observed in tumor spheroids without PS saturation based on FLI of DiR, whereas 42±4% of DiR molecules delivered by the same micelles were detected in tumor spheroids under the condition of PS saturation, indicating decreased DiR accumulation by 2.4-fold in a competitive binding test. Regardless of PS saturation, FLI of DiR molecules delivered by methoxy-PEG-*b*-PCL micelles (55–60%) was not statistically different.

### *Ex vivo* Apoptosis Detection

**Figure 3:**
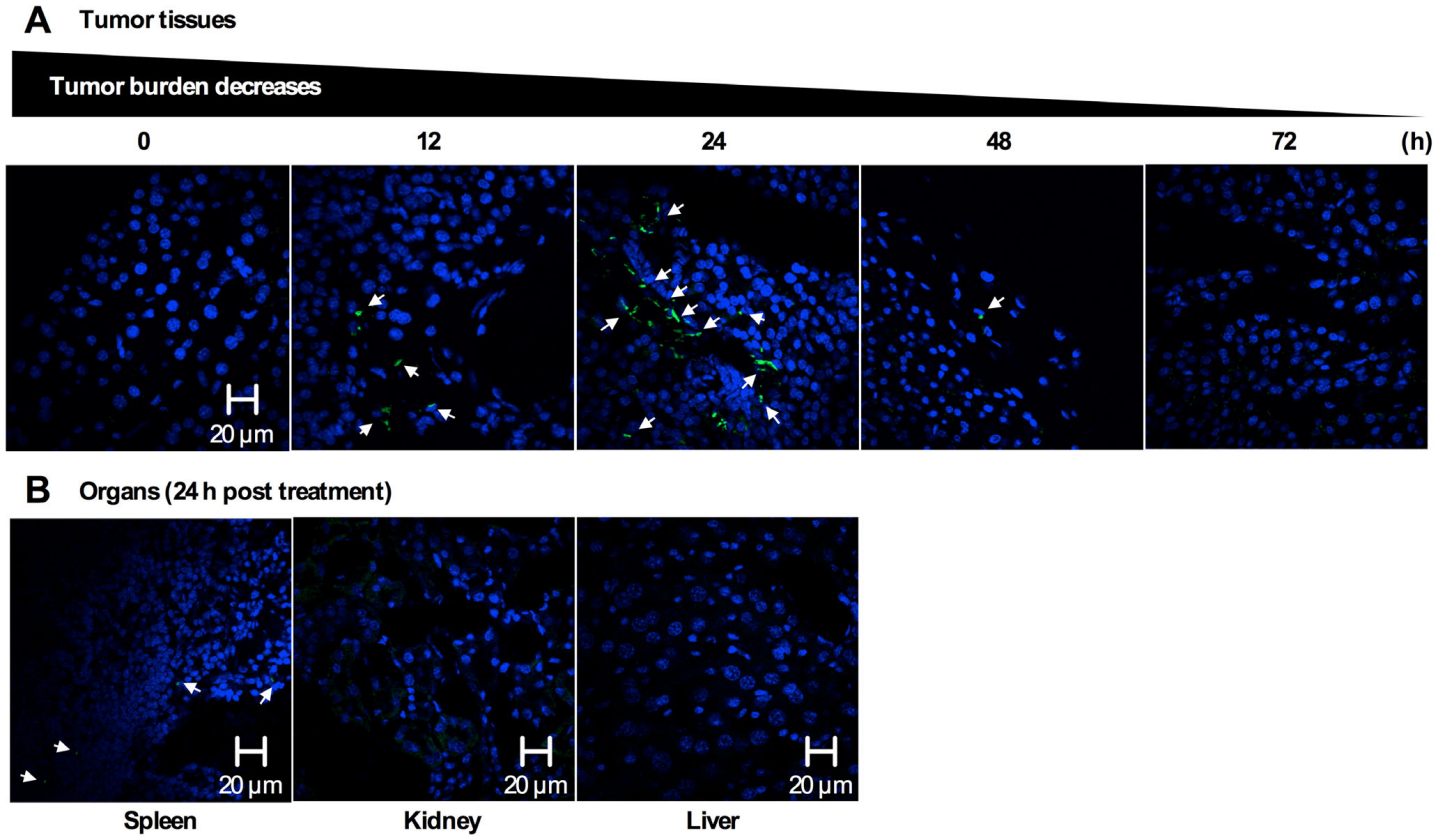
Apoptosis detection in tissue sections. (A) Laser scanning confocal microscopic images (60×magnification) of apoptosis by TUNEL in resected ES-2-luc ovarian tumor tissue at 0, 12, 24, 48, and 72 h post a single IV injection of PEG-*b*-PCL micelles carrying PTX, CYP, and GSP at 30, 30, and 30 mg/kg (B) Laser scanning confocal microscopic images (60×magnification) of apoptosis by TUNEL in resected spleen, kidney, and liver at 24 h post a single IV injection of PEG-*b*-PCL micelles carrying PTX, CYP, and GSP at 30, 30, and 30 mg/kg. DNA fragmentation in apoptotic cells are in green, and nuclei of cells are in blue (DAPI).

Evidence for apoptosis induction at tumor tissues after a single IP injection of PEG-*b*-PCL micelles carrying PTX, CYP, and GSP to ES-2-luc-bearing xenograft (on day 7 post IP ES-2-luc cell inoculation) was obtained from resected tumor tissues, which were collected at 12, 24, 48, and 72 h after an IP injection of PEG-*b*-PCL micelles carrying PTX, CYP, and GSP at 30, 30, and 30 mg/kg, respectively, sectioned into 10 µm, and visualized by confocal microscope. In Figure 3, a few green colored-DNA fragments in apoptotic cells appeared at tumor tissues at 12 h, more green dots were clearly visible at 24 h, and then there were faint signs of DNA fragmentation were present 48 h post treatment. Apoptotic cells at the kidney and liver were not visible, but a few apoptotic cells were observed at the spleen at 24 h post treatment.

### Apoptosis-targeting Efficacy of GFNFRLKAGAKIRFGS-PEG-*b*-PCL Micelles Carrying DiR *in vivo*

**Figure 4:**
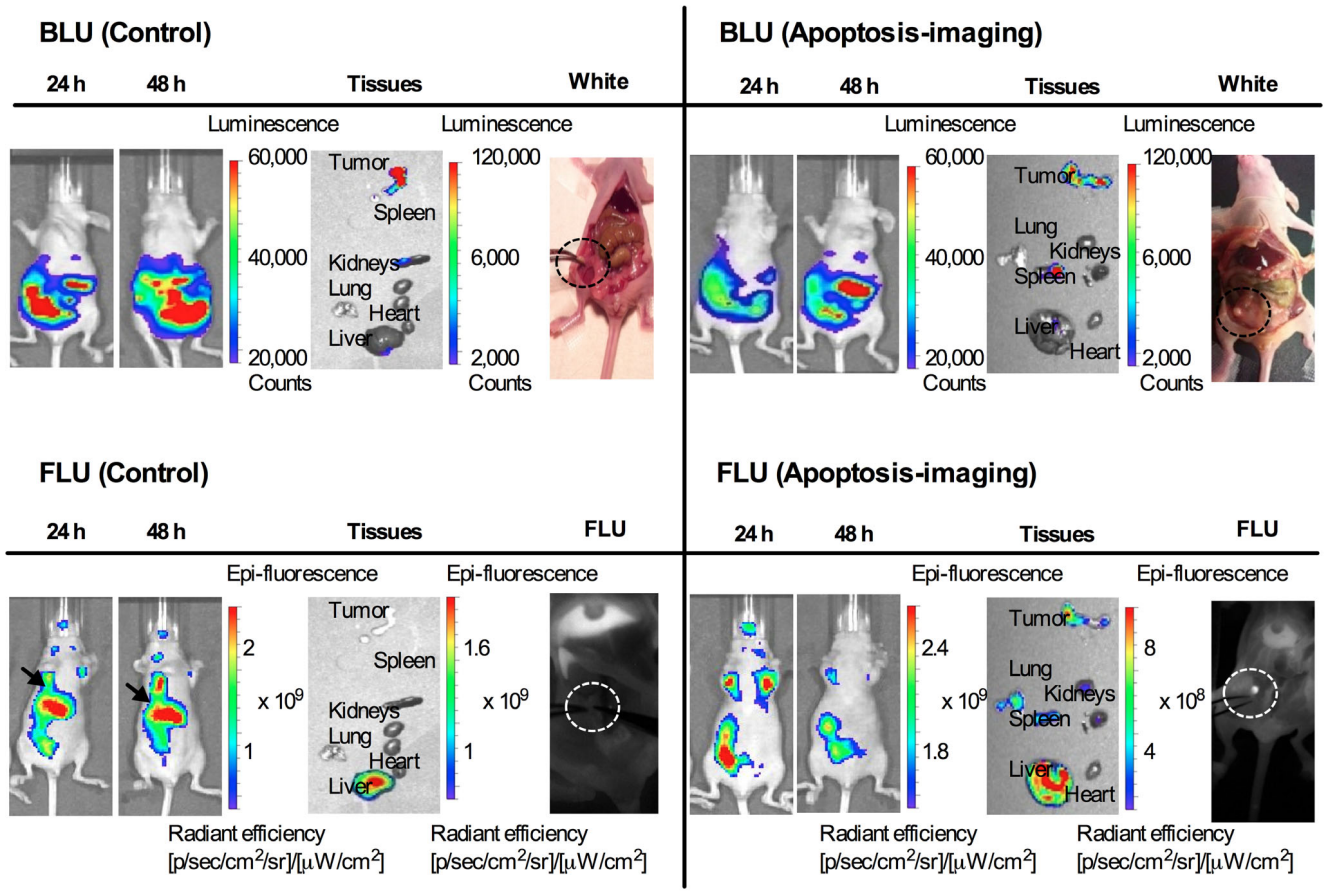
Assessment of apoptosis imaging *in vivo* and *ex vivo.* Whole-body and excised tissues in bioluminescence images of ES-2-luc-bearing mice and those in fluorescence images of same animals obtained with Xenogen IVIS 200 Series. White-light *vs*. fluorescence imagings in mid-line incised ES-2-luc-bearing mice obtained with Fluobeam 800. (A) Representative fluorescence and bioluminescence images of ES-2-luc-bearing mouse injected IP with empty PEG-*b*-PCL micelles followed by an IV injection of methoxy-PEG-*b*-PCL micelles carrying DiR at 250 µg/kg as a control (left hand side) and those of ES-2-luc-bearing xenograft model injected IP with PEG-*b*-PCL micelles carrying PTX, CYP, and GSP at 30, 30, and 30 mg/kg followed by an IV injection of PS-targeting PEG-*b*-PCL micelles carrying DiR at 250 µg/kg as an experimental group (right hand side) at 24 and 48 h after termination of treatments.

NIR fluorescence imaging of DiR delivered by either methoxy-PEG-*b*-PCL or apoptosis-targeting PEG-*b*-PCL micelles carrying DiR was observed longitudinally in groups of four animals (Figure 4). Subsequently, peritoneal dissemination of ES-2-luc tumor cells was monitored in identical animals, detecting bioluminescence signal over the peritoneal cavity. A group of animals was treated with PEG-*b*-PCL micelles carrying PTX, CYP, and GSP at 30, 30, and 30 mg/kg, respectively, (delivered IP on day 7 post ES-2-luc cell inoculation) followed by apoptosis-targeting PEG-*b*-PCL micelles carrying DiR at 100 nM (delivered IV on day 8); as shown on the right hand side in Figure 4 (an experimental group), whole-body bioluminescence of ES-2-luc tumor cells is shown along with the image of DiR delivered by apoptosis-targeting PEG-*b*-PCL micelles, shown in whole-body NIR fluorescence images at 24 h after an IV injection of apoptosis-targeting PEG-*b*-PCL micelles carrying DiR. In a control group of animals treated with empty PEG-*b*-PCL micelles followed by methoxy-PEG-*b*-PCL micelles carrying DiR, the distribution of DiR in fluorescence images showed lower correspondence with the peritoneal dissemination of ES-2-luc tumor cells from bioluminescence images with the strongest bioluminescence signal observed in liver (black arrows) of animals. Tumor tissues collected from an experimental group of animals were clearly color-coded in both bioluminescence and fluorescence images. Tumor tissues collected from a control group of animals lacked fluorescence in images. Additionally, it was confirmed by Fluobeam 800, a portable hand-held 2-D NIR fluorescence imaging system that a visually palpable tumor tissue under white-light was also clearly illuminated in gray-scale NIR fluorescence images of animals treated with apoptosis-targeting PEG-*b*-PCL micelles (an experimental group) after the NIR laser of Fluobeam 800 was on (dotted circles); however, a palpable tumor tissue was not visible or distinguishable from normal tissues in NIR fluorescence images of animals in the control group under the laser of Fluobeam 800.

**Figure 5:**
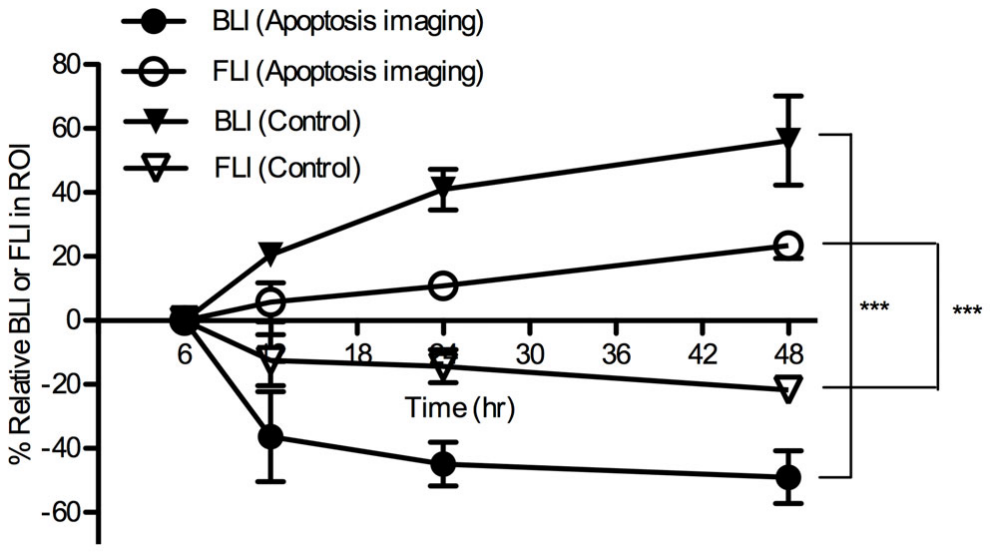
Relative BLI and FLI of ROIs in noninvasive whole-body images of ES-2-luc-bearing mouse in a time-dependent manner (*** <0.001).

From noninvasive bioluminescence imaging, quantitative bioluminescence intensity (BLI) in ROI (region of interest: tumor tissues in the abdomen of animals) is presented longitudinally in Figure 5. Two days after termination of treatment, %BLI in ROI (%change in BLI of ROI starting at 6 h after after an IV injection of apoptosis-targeting PEG-*b*-PCL micelles carrying DiR) increased rapidly up to *ca*. 60% but %FLI in ROI decreased to *ca*. −20% in control mice. In contrast, in the experimental group, %BLI in ROI decreased to *ca*. −50% but %FLI increased to *ca*. 20%.

### Real-time NIR Fluorescence Image-guided Surgery in IP Ovarian Cancer Model

**Figure 6:**
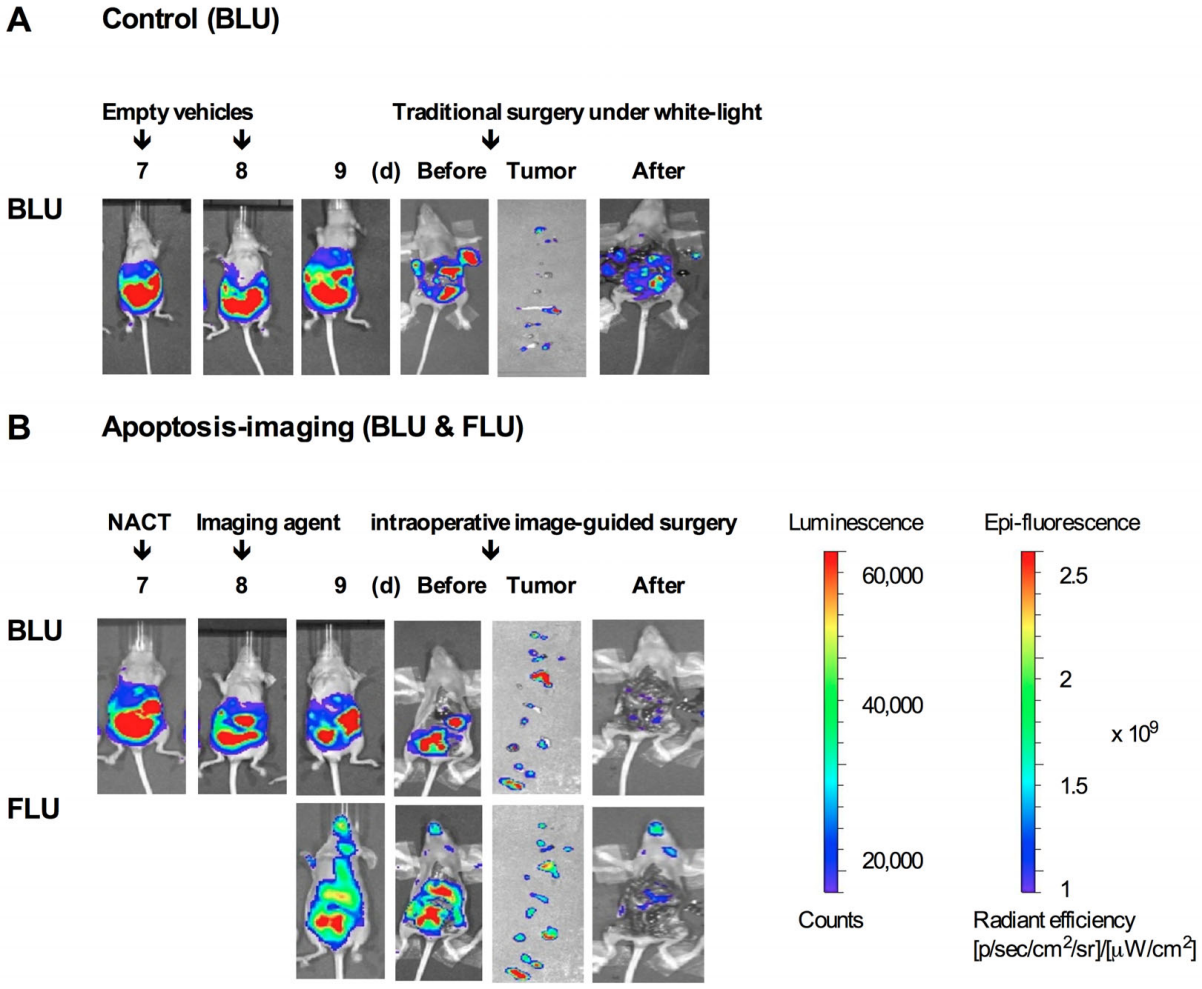
Assessment of intraoperative apoptosis imaging. Whole-body, excised tumor tissues, and carcass (before and after surgery) in bioluminescence and fluorescence images of four individual ES-2-luc-bearing xenograft animals obtained with Xenogen IVIS 200 Series. (A) ES-2-luc-bearing mice injected IP with empty PEG-*b*-PCL micelles followed by empty apoptosis-targeting PEG-*b*-PCL micelles as a control. (B) ES-2-luc-bearing mice injected IP with PEG-*b*-PCL micelles with PTX, CYP, and GSP at 30, 30, and 30 mg/kg followed by an IV injection of PS-targeting PEG-*b*-PCL micelles carrying DiR at 250 µg/kg as an experimental group.

ES-2-luc-bearing mice received an IP injection of either empty PEG-*b*-PCL micelles or PEG-*b*-PCL micelles carrying PTX, CYP, and GSP at 30, 30, and 30 mg/kg, respectively, on day 7, followed by a subsequent IV injection of either empty apoptosis-targeting PEG-*b*-PCL micelles or apoptosis-targeting PEG-*b*-PCL micelles carrying DiR on day 8 post IP cell inoculation. On day 9, all animals were sacrificed, and the abdominal cavity was opened. Surgical tumor resection of the visually palpable tumor-like tissues was performed under white-light in control mice (traditional surgery). Resection of the fluorescence-positive (FLU^+^) tumor-like tissues was performed under real-time NIR guidance using the Fluobeam 800 fluorescence imaging system (intraoperative fluorescence image-guided surgery). Bioluminescence images were obtained of mice before incision, mice after laparotomy incision, mice after debulking surgery, and of collected tumor-like tissues for a control group (*n* = 4) in Figure 6A; bioluminescence and fluorescence images of the same were for an experimental group (*n* = 4) in Figure 6B. Not all dissected tumor-like tissues by traditional surgery under white-light were bioluminescence-positive (LUC^+^) tissues and only some of dissected tumor-like tissues were genuine luciferase-expressing tumor tissues. The large number of tumors left undissected in the carcass after surgical tumor resection under white-light is shown in Figure 6A. In contrast, most of tumor-like tissues dissected with the guidance of intraoperative NIR fluorescence imaging were LUC^+^ and good correlation was observed between fluorescence and bioluminescence patterns in the collected tumor tissues (Figure 6B). Substantially smaller amounts of LUC^+^ and FLU^+^ tissues were left undissected in the carcass after real-time NIR image-guided surgery.

**Figure 7:**
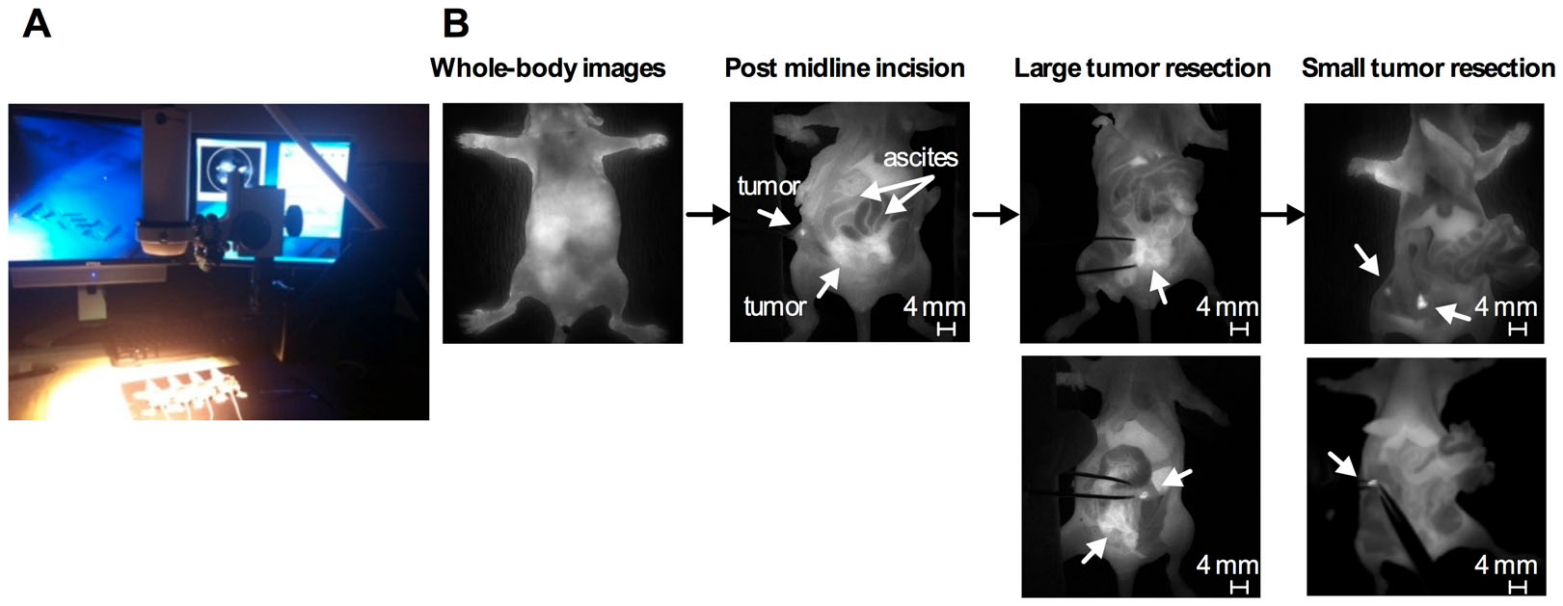
Surgical procedure of ES-2-luc bearing mice guided by intraoperative NIR fluorescence imaging of apoptosis. (A) Experimental conduct of animal surgery using Fluobeam. (B) Fluorescence images of carcass of ES-2-luc-bearing mice captured by Fluobeam during the peritoneal exploration and surgery.

**Figure 8:**
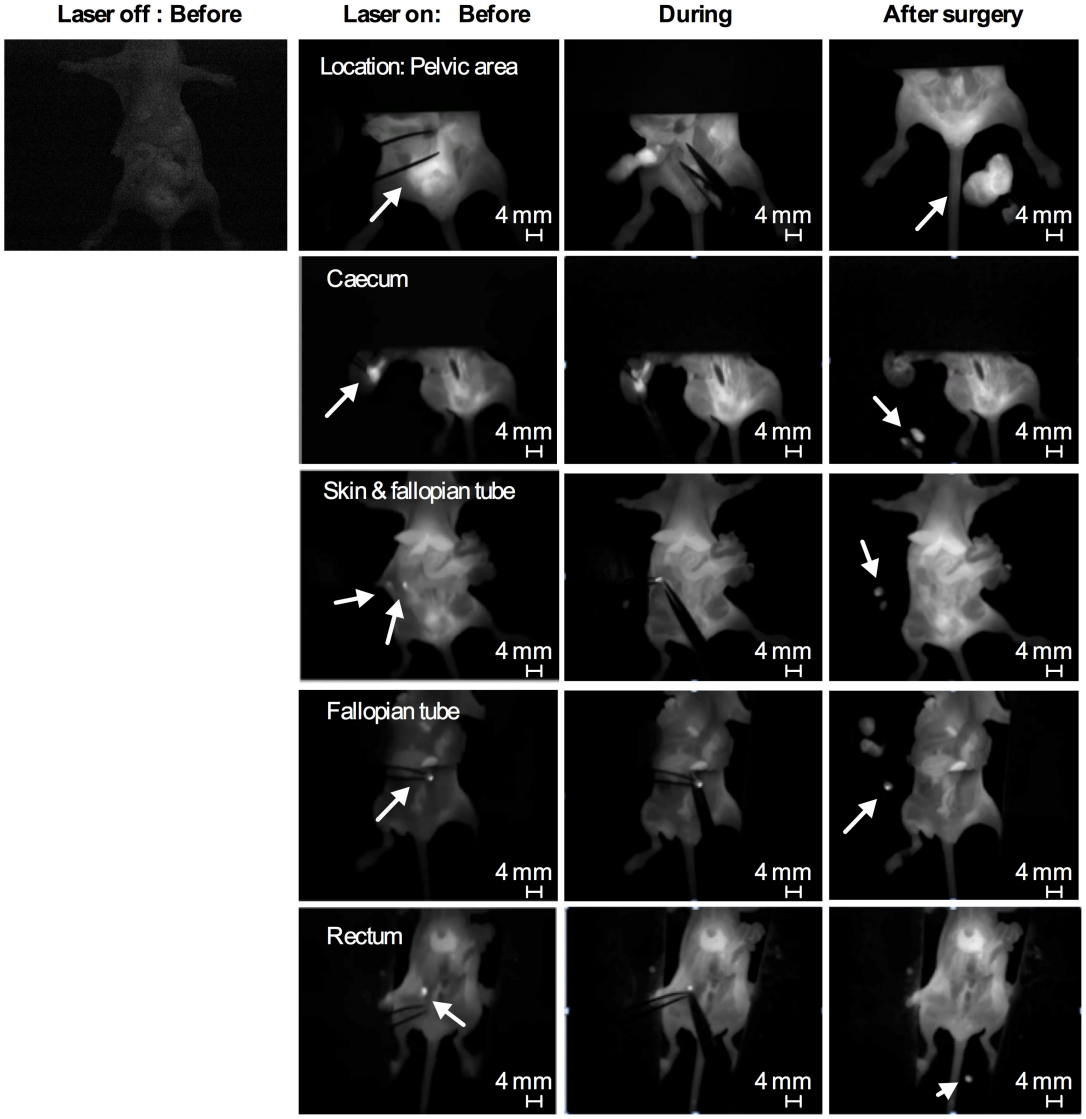
Fluorescence images of carcass of ES-2-luc-bearing mice at different surgical stages and locations obtained by Fluobeam.

The surgical procedure guided by intraoperative grey-scale NIR fluorescence images using Fluobeam 800 in ES-2-bearing animals (an experimental group) is shown in Figure 7; apoptosis-targeting PEG-*b*-PCL micelles carrying DiR allowed the detection of FLU^+^ (white in color) large ovarian tumor tissues and FLU^+^ ascites in open peritoneum, noting that BLU^+^ cancer cells in ascites are also fluorescent. Following thorough exploration and surgical removal of large FLU^+^ tumor tissues and FLU^+^ ascites, smaller tumor nodules are visible for resection from peritoneum. Representative intraoperative images obtained before, during, and after interval debulking surgery in ES-2-bearing animals in an experimental group are presented in Figure 8; FLU^+^ tissues located in pelvic area, caecum, skin, fallopian tube, and rectum were observed under Fluobeam 800. Real-time NIR fluorescence imaging guidance permitted detection of tumors *ca*. 1 mm in diameter (Figures 7 and 8). Real-time video clips collected from Fluobeam 800 are supplied as Supporting Information (Movies S1-4).

The amount of undissected Luc^+^ tissues left in carcasses before and after interval debulking surgery was quantified, and the number of total resected tissues and the number of LUC^+^ tissues were counted using visual observation and by bioluminescence imaging, respectively (Table 2). By comparing the number of LUC^+^ tissues with the number of total resected tissues, surgical accuracy could be estimated (equation 1: % no. of BLU^+^ tissues/no. of total resected tissues). Following traditional surgery, 33.5±16.6% of resected tissues were real LUC^+^ tumor tissues. Guided by intraoperative NIR fluorescence images, 91.7±8.6% of resected tissue were LUC^+^ tumor tissues. Surgical accuracy was also assessed by estimating %BLI of dissected tumors (equation 2: [BLI of ROI in mid-line incised body “before” surgery – BLI of ROI in dissected body “after” surgery]) divided by BLI of ROI in mid-line incised body “before” surgery. Traditional surgery removed 27.5±9.9% of LUC^+^ tissues and intraoperative NIR fluorescence image guidance resected 88.6±6.1% of LUC^+^ tissues from ES-2-luc-bearing mice.

**Table 2:** Evaluation of apoptosis-targeted interval debulking surgery in ES-2-luc-bearing xenograft model.

Group	% Accuracy Equation 1(*ex vivo* tissue-based)	% Accuracy Equation 2(*ex vivo* carcass-based)	Average operationtime (min)	PCI (BLI based)
**Experimental**	91.7±8.6	88.6±6.1	12.5±2.0	6.8±3.4
**Control**	33.5±16.6	27.5±9.9	9.2±1.3	26.5±8.1

**Figure 9:**
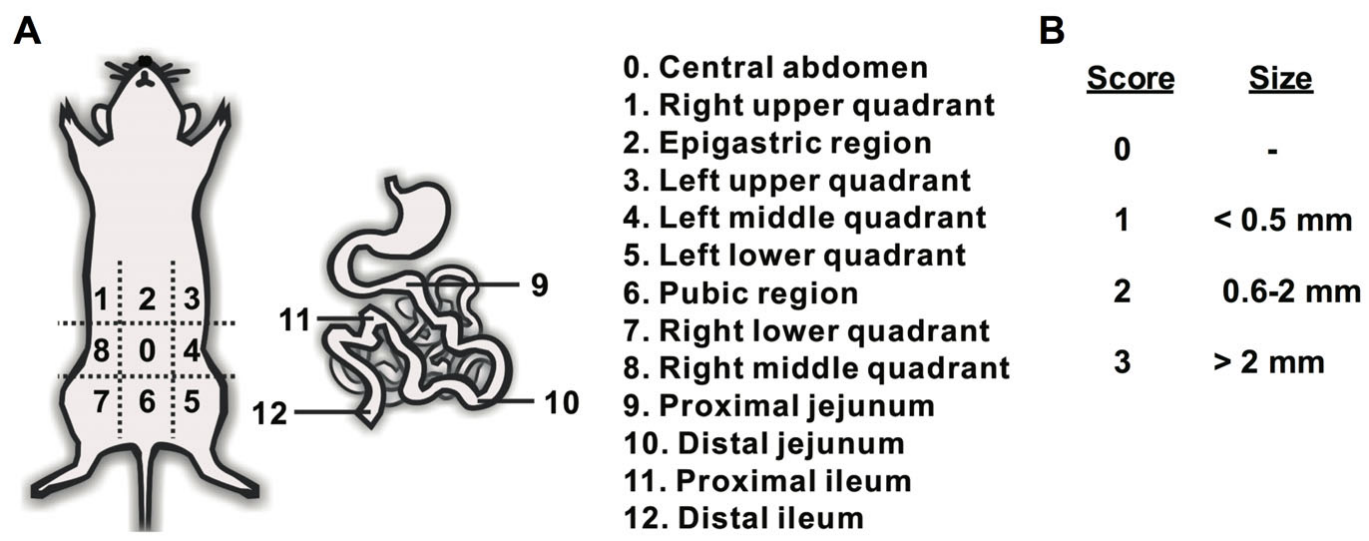
Illustration of Peritoneal Cancer Index (PCI) by Sugarbaker. (A) A composite score (0–3) of lesion size in abdomino-pelvic regions (0–12). (B) The scoring system of PCI adapted from Sugarbaker.

Average duration of interval debulking surgery was 9.2±1.3 min under white-light in traditional surgery and 12.5±2.0 min using the intraoperative NIR fluorescence imaging guidance. The median peritoneal cancer index (PCI) calculated using the scoring system shown in Figure 9 was 26.5±8.1 after traditional surgery and 6.8±3.4 after surgery guided by real-time NIR fluorescence images.

## Discussion

In preclinical and clinical trials, there are four factors to consider in determining the success of intraoperative NIR image-guided surgery: (1) targeting agent, (2) disease type, (3) surgical intent, and (4) imaging equipment [26]. Our study demonstrated that a combined approach with IP NACT and real-time NIR fluorescence image-guided interval debulking surgery may represent an improved strategy for ovarian cancer, addressing the aforementioned four factors as follows. (1) Targeting agents: several NIR fluorescence probes and fluorescence agent-containing or -conjugated nanoparticles have been developed for a tumor-targeted imaging purposes, *eg*. Cy5-labeled RAFT-c(RGDfK)_4,_ Cy5.5-labled glycol chitosan nanoparticles, and GRGDS-PEG-*b*-PCL micelles containing DiI [23], [27], [28]. Selection of tumor targets in tumors impacts the surgical treatment because the surgeon completely relies on images to be visible in the surgical field. In this study, therapy-induced apoptosis was chosen as a target for intraoperative NIR fluorescence imaging and a strategy of targeting apoptosis at tumor tissues using apoptosis-targeting peptide (GFNFRLKAGAKIRFGS)-conjugated PEG-*b*-PCL micelles containing the NIR fluorescence probe DiR was validated in a preclinical mouse model of ovarian cancer debulking. Li and colleagues identified a 14-mer peptide, FNFRLKAGAKIRFG, which demonstrated nanomolar binding affinity to PS in apoptotic tumors for therapy-induced apoptosis imaging purpose [29]. In our study, a FNFRLKAGAKIRFG-containing linear peptide, GFNFRLKAGAKIRFGS (G and S were added to enhance serum stability of FNFRLKAGAKIRFG residues after conjugation), was conjugated on the surface of aldehyde-PEG-*b*-PCL micelles for serum stability and apoptosis-targeted delivery of a NIR fluorescence imaging probe. DiR-incorporated GFNFRLKAGAKIRFGS-PEG-*b*-PCL micelles (named as apoptosis-targeting PEG-*b*-PCL micelles) prepared by a solvent evaporation method obtained similar physical characteristics of DiR-incorporated methoxy-PEG-*b*-PCL micelles, *eg*. DiR loading capacity at *ca.* 2% and the t_1/2_ value for *in vitro* DiR release at *ca.* 2 h, but 1.8-fold larger z-average diameter of apoptosis-targeting PEG-*b*-PCL micelles carrying DiR at *ca.* 80 nm. Despite their slightly enlarged particle size, there were no signs of uneven size distribution or aggregation of apoptosis-targeting PEG-*b*-PCL micelles in aqueous solution (PDI 0.1) as shown in Table 1, enabling safe IV injection for *in vivo* studies without rapid renal clearance (typically problematic for a low molecular weight peptides) or severe hepatic uptake of aggregates. GFNFRLKAGAKIRFGS, maintained PS-selective binding affinity after decorating the surface of aldehyde-PEG-*b*-PCL micelles and apoptosis-targeting PEG-*b*-PCL micelles adsorbed selectively onto PS-coated wells and apoptosis-induced ES-2 ovarian tumor spheroids *in vitro* (Figure 4). Apoptosis-targeting PEG-*b*-PCL micelles preferentially delivered DiR molecules preferably to apoptosis-induced ES-2-luc ovarian tumor spheroids with 2.4-fold increased DiR uptake over pre-saturated ES-2-luc ovarian tumor spheroids. Apoptosis-targeting PEG-*b*-PCL micelles carrying DiR have potential as a promising NIR fluorescence imaging agent for intraoperative surgical guidance in oncology, as they satisfy the major needs of NIR fluorescence imaging agents (facile preparation, the ability to be injectable as nanomaterials, enhanced aqueous solubility of highly lipophilic imaging probe, strong light absorbance in the NIR window, and most importantly, active tumor targeting).

(2) Disease type: a tandem of PEG-*b*-PCL micelles, PEG-*b*-PCL micelles carrying PTX, CYP, and GSP for NACT-induced apoptosis and apoptosis-targeting PEG-*b*-PCL micelles carrying DiR for optical imaging purpose, was tested in metastatic human ovarian cancer (ES-2-luc)-bearing animal model categorized as undifferentiated type in ovarian carcinomas. The undifferentiated carcinoma is defined by the absence of distinctive histological features or only small foci of differentiation, and is known as clinically aggressive neoplasm accounting for 4–5% of all primary ovarian malignancies [30], [31]. ES-2-luc cancer cells inoculated in the peritoneal cavity tend to widely spread in the abdominal peritoneum and rapidly invade ovaries, fallopian tubes, pelvic structures (uterus, bladder, and rectum), and intestines (Figure S2), leading to 100% animal death within 4 weeks after IP inoculation [21]. Due to the aggressive nature of peritoneal malignancies, successful treatment of metastatic peritoneal ovarian cancer is challenging and requires aggressive debulking surgery [3].

(3) Surgical intent: because of the number and broad distribution of tumors, surgical resection of aggressively metastatic ovarian cancer requires a large incision, thorough explorations, and precise tumor resection with minimization of injury to involved organs. It is therefore important to clearly delineate tumor margins and differentiate highly undistinguishable lesions from normal tissues. Confocal microscopic images of collected tissues demonstrated that a single IP injection of PEG-*b*-PCL micelles carrying PTX, CYP, and GSP at 30, 30, and 30 mg/kg, respectively, in ES-2-luc-bearing animals increased the level of apoptosis in tumor tissues over time, reaching to the peak apoptosis level on day 1 post drug treatment. The subsequent decrease in the number of DNA fragments was presumably due to DNA fragments of apoptotic cells engulfed by macrophages. No apoptosis was observed in the kidney and liver but, a few DNA fragments of apoptotic cells were found in the spleen on day 1: as ES-2-luc ovarian cancer cells often travel to spleen and pancreas, it is possible that DNA fragments might be induced in metastatic tumor tissues residing in spleen. When apoptosis induction was optimized at 24 h after an IP injection of PEG-*b*-PCL micelles carrying PTX, CYP, and GSP in ES-2-luc-bearing animals, PEG-*b*-PCL micelles carrying DiR were injected *via* IV route. Some correlation was observed between NIR fluorescence signal (from DiR molecules accumulating at tumor tissues) and BLI of tumor tissues on bioluminescence imaging 24 h after an IV injection of apoptosis-targeting PEG-*b*-PCL micelles carrying DiR (Figure 4). However, methoxy-PEG-*b*-PCL micelles carrying DiR were observed mostly in the liver, indicating that passive targeting by the EPR effect was not sufficient to deliver DiR-incorporated methoxy-PEG-*b*-PCL micelles to metastatic tumor tissues disseminated in the peritoneal cavity. Moreover, a tumor priming strategy achieved by IP injection of PEG-*b*-PCL micelles carrying PTX, CYP, and GSP followed by an IV injection of methoxy-PEG-*b*-PCL micelles carrying DiR, was also insufficient to deliver DiR-incorporated methoxy-PEG-*b*-PCL micelles to metastatic tumor tissues in our ES-2-luc-bearing xenograft model (data not shown). As metastatic ovarian tumor tissues are deficient in blood supply and spread discontiguously along the peritoneal surfaces, microenvironment of metastatic ovarian tumors may impede delivery of agents by EPR effect. The present study demonstrates that apoptosis-targeting PEG-*b*-PCL micelles carrying DiR improved delivery of DiR molecules to metastatic ovarian tumor tissues with a 1.5-fold stronger FLI in collected tumor tissues compared to passively-targeted DiR using methoxy-PEG-*b*-PCL micelles at 48 h after IV injection. To illustrate the potential utility of our observations, small tumor tissues that were routinely left undissected near the left fallopian tube that were not visible by DiR-incorporated methoxy-PEG-*b*-PCL micelles under Fluobeam 800, were clearly visible by apoptosis-targeting PEG-*b*-PCL micelles carrying DiR under the same condition with Fluobeam 800 (Figure 4). In this study, the surgical intention for undifferentiated ovarian cancer at advanced stage is certainly to eradicate ovarian tumors after complete interval debulking surgery with minimum residual tumors (<1 cm in size of residual tumors) guided by intraoperative NIR fluorescence imaging [1].

(4) Imaging equipment: Fluobeam 800 provided real-time gray-scale images after NIR excitation of surgical targets and allowed for acquisition of both static and real-time images of objects (Figures 8 and 9, and Movies S1-4). The real-time feedback of NIR fluorescence images during operative exploration can maximize the practical benefit of NIR fluorescence imaging compared to wide-field whole-body epifluorescence illumination approach by minimizing concerns regarding depth of detection and high autofluorescence from skin, as a large abdominal incision is necessary for the surgical process in peritoneal malignancies. In the experimental group, IP NACT followed by interval debulking surgery with a support of real-time NIR fluorescence images, resulted in *ca.* 90% debulking of ES-2-luc tumor tissue, whereas *ca.* 30% of ES-2-luc tumor tissues were removed in the control group of traditional debulking surgery (IP and IV injections of empty vehicles) under white-light (Figure 7 and Table 2). The surgical superiority of real-time NIR fluorescence image-guided surgery was also demonstrated using the peritoneal cancer index (PCI), an indicator of distribution and size of residual tumors, adapted from Sugarbaker (7 *vs.* 27) [3]. A modified PCI scoring system was applied in our study to translate the scoring system from human to mouse scale. Unlike results reported by Coll and colleagues, in which the operation time of surgical tumor resection was reduced with the use of Fluobeam 800 in peritoneal adenocarcinoma-bearing mouse model, intraoperative fluorescence NIR image-guidance did not reduce the duration of surgery compared to traditional white-light surgery (13 min *vs.* 9 min). In our experience, it was evident that the additional tumors visualized by real-time NIR fluorescence (not seen under white light) necessitated longer operation time, resulting in more optical tumor debulking. Surprisingly, real-time videos recorded by Fluobeam 800 show that the NIR fluorescence signal of DiR molecules at tumor tissues permitted identification of tumors as small as 1 mm in diameter and as large as 5 mm in diameter. The heightened sensitive tumor detection could greatly improve the efficacy of surgical therapy for peritoneal metastases, as optical tumor debulking ideally requires minimum residual tumors <1 cm [32]. It is expected that improved detection of submillimeter sized-tumor tissues could enhance the delivery of surgical therapy and ultimately improve survival [7]. Future work will focus on the evaluation of the current treatment strategy in a metastatic ovarian cancer-bearing rat model and to assess the feasibility of its apoptosis induction, improved tumor delineation, and efficient surgical tumor removal to prolong overall survival.

## Conclusions

Optical NIR fluorescence imaging has shown potential for intraoperative surgical guidance in ovarian cancer beyond preoperative radiological imaging and visual inspection or palpation of tumors under white-light illumination. A successful strategy of ovarian cancer management requires a combination of aggressive surgical therapy with chemotherapy.

In this study, a treatment strategy coupling NACT and an interval debulking surgery guided by intraoperative apoptosis-targeted NIR fluorescence imaging using a tandem of administrated PEG-*b*-PCL micelles resulted in induction of apoptosis in tumor tissues, accurate delineation of tumor tissues in NIR fluorescence images, and ultimately, improved surgical accuracy and outcome in an ES-2-luc-bearing xenograft model of ovarian cancer. The unique approach employing PEG-*b*-PCL micelles may be seamlessly integrated into ovarian cancer surgery, enhancing visualization of tumor tissues and providing valuable guidance using intraoperative NIR fluorescence imaging for surgical oncology.

## Supporting Information

Figure S1 **Synthesis of the GFNFRLKAGAKIRFGS-PEG-** ***b*** **-PCL.**
(TIFF)

Figure S2 **Carcass of ES-2-luc-bearing xenograft model on day 25 post IP inoculation of ES-luc cells (1×10^6^ cells/animal).**
(TIFF)

Movie S1 **Real-time fluorescence NIR images of ES-2-luc-bearing xenograft models during surgical tumor resection (right lower quadrant).** ES-2-luc-bearing xenograft models were treated with an IP injection of PEG-*b*-PCL micelles carrying PTX, CYP, and GSP on day 7, IV injection of apoptosis-targeting PEG-*b*-PCL micelles carrying DiR on day 8, and surgical tumor resection on day 9 post ES-2-luc cell inoculation.
(MP4)

Movie S2 **Real-time fluorescence NIR images of ES-2-luc-bearing xenograft models during surgical tumor resection (skin and fallopian tube).**
(MP4)

Movie S3 **Real-time fluorescence NIR images of ES-2-luc-bearing xenograft models during surgical tumor resection (stomach).**
(MP4)

Movie S4 **Real-time fluorescence NIR images of ES-2-luc-bearing xenograft models during surgical tumor resection (fallopian tube).**
(MP4)

## References

[1] Schorge JO, McCann C, Del Carmen MG (2010) Surgical debulking of ovarian cancer: what difference does it make? Rev Obstet Gynecol 3: 111–117. 21364862 PMC3046749

[2] Kim A, Ueda Y, Naka T, Enomoto T (2012) Therapeutic strategies in epithelial ovarian cancer. J Exp Clin Cancer Res 31: 14. 22330607 10.1186/1756-9966-31-14 PMC3309949

[3] Sugarbaker PH (1999) Management of peritoneal-surface malignancy: the surgeon’s role. Langenbecks Arch Surg 384: 576–587. 10654274 10.1007/s004230050246

[4] Hirche C, Engel H, Kolios L, Cognie J, Hunerbein M, et al.. (2012) An Experimental Study to Evaluate the Fluobeam 800 Imaging System for Fluorescence-Guided Lymphatic Imaging and Sentinel Node Biopsy. Surg Innov. 10.1177/1553350612468962 23275469

[5] Keramidas M, Josserand V, Righini CA, Wenk C, Faure C, et al (2010) Intraoperative near infrared image-guided surgery for peritoneal carcinomatosis in a preclinical experimental model. Br J Surg 97: 737–743. 20309948 10.1002/bjs.6986

[6] Troyan SL, Kianzad V, Gibbs-Strauss SL, Gioux S, Matsui A, et al (2009) The FLARE intraoperative near-infrared fluorescence imaging system: a first-in-human clinical trial in breast cancer sentinel lymph node mapping. Ann Surg Oncol 16: 2943–2952. 19582506 10.1245/s10434-009-0594-2 PMC2772055

[7] van Dam GM, Themelis G, Crane LM, Harlaar NJ, Pleijhuis RG, et al (2011) Intraoperative tumor-specific fluorescence imaging in ovarian cancer by folate receptor-alpha targeting: first in-human results. Nat Med 17: 1315–1319. 21926976 10.1038/nm.2472

[8] Keereweer S, Kerrebijn JD, van Driel PB, Xie B, Kaijzel EL, et al (2011) Optical image guided surgery–where do we stand? Mol Imaging Biol 13: 199–207. 20617389 10.1007/s11307-010-0373-2 PMC3051067

[9] Altinoglu EI, Russin TJ, Kaiser JM, Barth BM, Eklund PC, et al (2008) Near-Infrared Emitting Fluorophore-Doped Calcium Phosphate Nanoparticles for In Vivo Imaging of Human Breast Cancer. ACS Nano 2: 2075–2084. 19206454 10.1021/nn800448r

[10] Gunasekera UA, Pankhurst QA, Douek M (2009) Imaging applications of nanotechnology in cancer. Target Oncol 4: 169–181. 19876702 10.1007/s11523-009-0118-9

[11] Janib SM, Moses AS, MacKay JA (2010) Imaging and drug delivery using theranostic nanoparticles. Adv Drug Deliv Rev 62: 1052–1063. 20709124 10.1016/j.addr.2010.08.004 PMC3769170

[12] Koo OM, Rubinstein I, Onyuksel H (2005) Role of nanotechnology in targeted drug delivery and imaging: a concise review. Nanomedicine 1: 193–212. 17292079 10.1016/j.nano.2005.06.004

[13] Kumar R, Roy I, Hulchanskyy TY, Goswami LN, Bonoiu AC, et al (2008) Covalently dye linked, surface-controlled, and bioconjugated organically modified silica nanoparticles as targeted probes for optical imaging. ACS Nano 2: 449–456. 19206569 10.1021/nn700370b

[14] Yokoyama M (2010) Polymeric micelles as a new drug carrier system and their required considerations for clinical trials. Expert Opin Drug Deliv 7: 145–158. 20095939 10.1517/17425240903436479

[15] Kim K, Lee M, Park H, Kim JH, Kim S, et al (2006) Cell-permeable and biocompatible polymeric nanoparticles for apoptosis imaging. Journal of the American Chemical Society 128: 3490–3491. 16536501 10.1021/ja057712f

[16] Lee S, Choi KY, Chung H, Ryu JH, Lee A, et al (2011) Real Time, High Resolution Video Imaging of Apoptosis in Single Cells with a Polymeric Nanoprobe. Bioconjugate Chemistry 22: 125–131. 21218786 10.1021/bc1004119

[17] Bae YH (2011) Apoptosis-targeted drug delivery. J Control Release 154: 213. 21906531 10.1016/j.jconrel.2011.08.024

[18] Schutters K, Reutelingsperger C (2010) Phosphatidylserine targeting for diagnosis and treatment of human diseases. Apoptosis 15: 1072–1082. 20440562 10.1007/s10495-010-0503-y PMC2929432

[19] Cho H, Indig GL, Weichert J, Shin HC, Kwon GS (2012) In vivo cancer imaging by poly(ethylene glycol)-b-poly(epsilon-caprolactone) micelles containing a near-infrared probe. Nanomedicine 8: 228–236. 21704593 10.1016/j.nano.2011.06.009 PMC3193583

[20] Cho H, Kwon GS (2011) Polymeric micelles for neoadjuvant cancer therapy and tumor primed optical imaging. ACS Nano 5: 8721–8729. 21999531 10.1021/nn202676u PMC3879117

[21] Cho H, Lai TC, Kwon GS (2012) Poly(ethylene glycol)-block-poly(epsilon-caprolactone) micelles for combination drug delivery: Evaluation of paclitaxel, cyclopamine and gossypol in intraperitoneal xenograft models of ovarian cancer. J Control Release 166: 1–9. 23246471 10.1016/j.jconrel.2012.12.005 PMC3565042

[22] Leblond F, Davis SC, Valdes PA, Pogue BW (2010) Pre-clinical whole-body fluorescence imaging: Review of instruments, methods and applications. J Photochem Photobiol B 98: 77 94. 10.1016/j.jphotobiol.2009.11.007 PMC3678966 20031443

[23] Xiong XB, Mahmud A, Uludag H, Lavasanifar A (2007) Conjugation of arginine-glycine aspartic acid peptides to poly(ethylene oxide)-b-poly(epsilon-caprolactone) micelles for enhanced intracellular drug delivery to metastatic tumor cells. Biomacromolecules 8: 874 884. 10.1021/bm060967g 17315946

[24] Kapty J, Banman S, Goping IS, Mercer JR (2012) Evaluation of Phosphatidylserine-Binding Peptides Targeting Apoptotic Cells. J Biomol Screen 17: 1293–1301. 22811476 10.1177/1087057112453313

[25] Igarashi K, Kaneda M, Yamaji A, Saido TC, Kikkawa U, et al (1995) A Novel Phosphatidylserine-Binding Peptide Motif Defined by an Antiidiotypic Monoclonal Antibody - Localization of Phosphatidylserine-Specific Binding-Sites on Protein-Kinase-C and Phosphatidylserine Decarboxylase. J Biol Chem 270: 29075–29078. 7493929 10.1074/jbc.270.49.29075

[26] Thurber GM, Figueiredo JL, Weissleder R (2010) Detection limits of intraoperative near infrared imaging for tumor resection. J Surg Oncol 102: 758–764. 20872807 10.1002/jso.21735 PMC3758114

[27] Jin ZH, Josserand V, Razkin J, Garanger E, Boturyn D, et al.. (2006) Noninvasive optical imaging of ovarian metastases using Cy5-labeled RAFT-c(-RGDfK-)4. Mol Imaging 5: 188 197. 16954034

[28] Park K, Kim JH, Nam YS, Lee S, Nam HY, et al (2007) Effect of polymer molecular weight on the tumor targeting characteristics of self-assembled glycol chitosan nanoparticles. J Control Release 122: 305–314. 17643545 10.1016/j.jconrel.2007.04.009

[29] Xiong C, Brewer K, Song S, Zhang R, Lu W, et al (2011) Peptide-based imaging agents targeting phosphatidylserine for the detection of apoptosis. J Med Chem 54: 1825–1835. 21348464 10.1021/jm101477d

[30] Lalwani N, Prasad SR, Vikram R, Shanbhogue AK, Huettner PC, et al (2011) Histologic, molecular, and cytogenetic features of ovarian cancers: implications for diagnosis and treatment. Radiographics 31: 625–646. 21571648 10.1148/rg.313105066

[31] Miyai K, Yamamoto S, Aida S, Shimazaki H, Takano M, et al (2010) Massive intra abdominal undifferentiated carcinoma derived from an endometrioid adenocarcinoma in a “normal-sized” ovary. Int J Gynecol Pathol 29: 321–327. 20567143 10.1097/PGP.0b013e3181c4f35f

[32] Frangioni JV (2008) New technologies for human cancer imaging. J Clin Oncol 26: 4012–4021. 18711192 10.1200/JCO.2007.14.3065 PMC2654310

