# Supplementary figures and images for: Polymeric Micelles for Apoptosis-Targeted Optical Imaging of Cancer and Intraoperative Surgical Guidance
Source: PLoS One. 2014 Feb 26;9(2):e89968. doi: 10.1371/journal.pone.0089968 (PMC3935963; doi:10.1371/journal.pone.0089968)

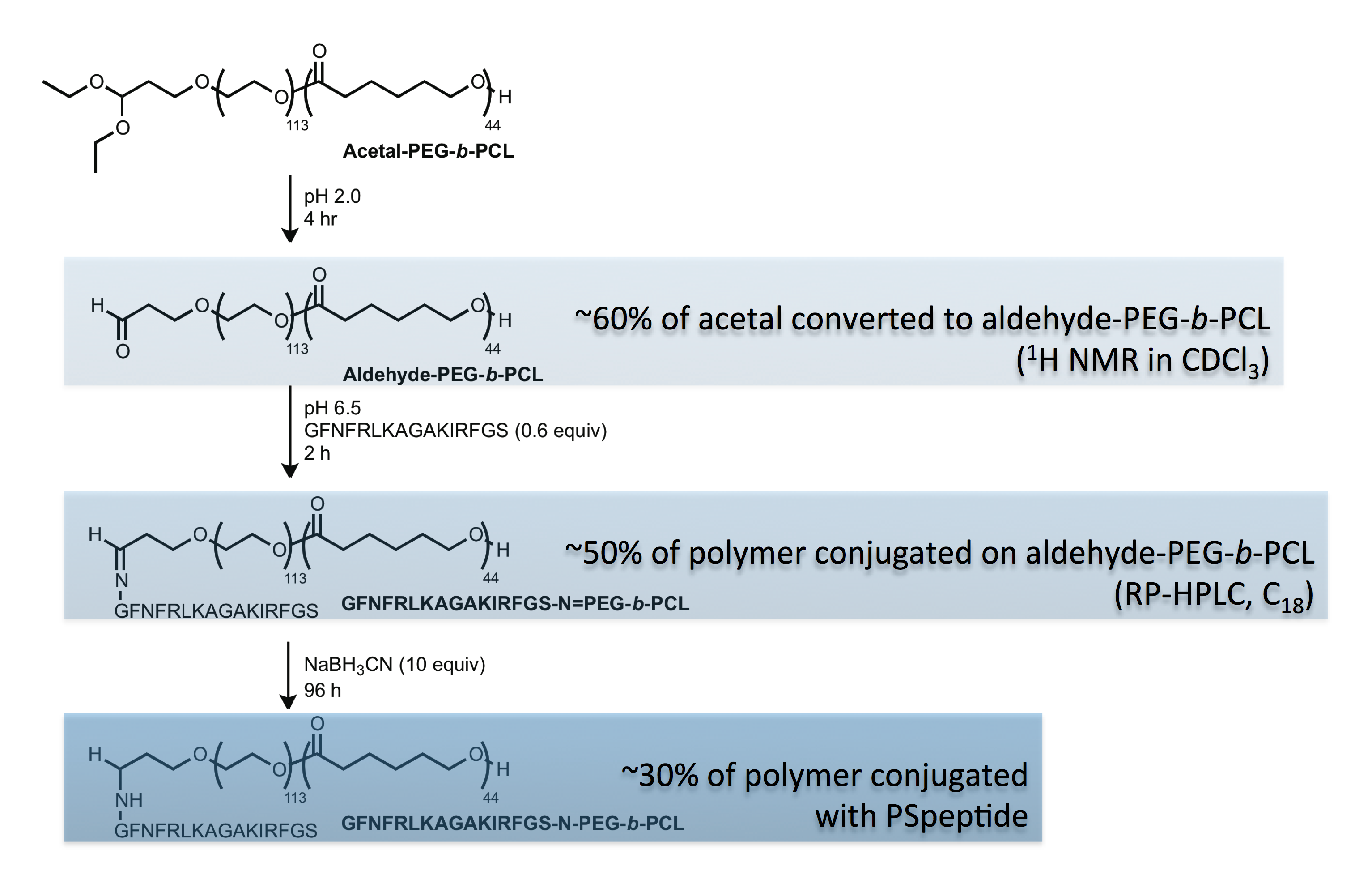

Supplement: Figure S1 — Synthesis of the GFNFRLKAGAKIRFGS-PEG- b -PCL. (TIFF) [file pone.0089968.s001.tiff]

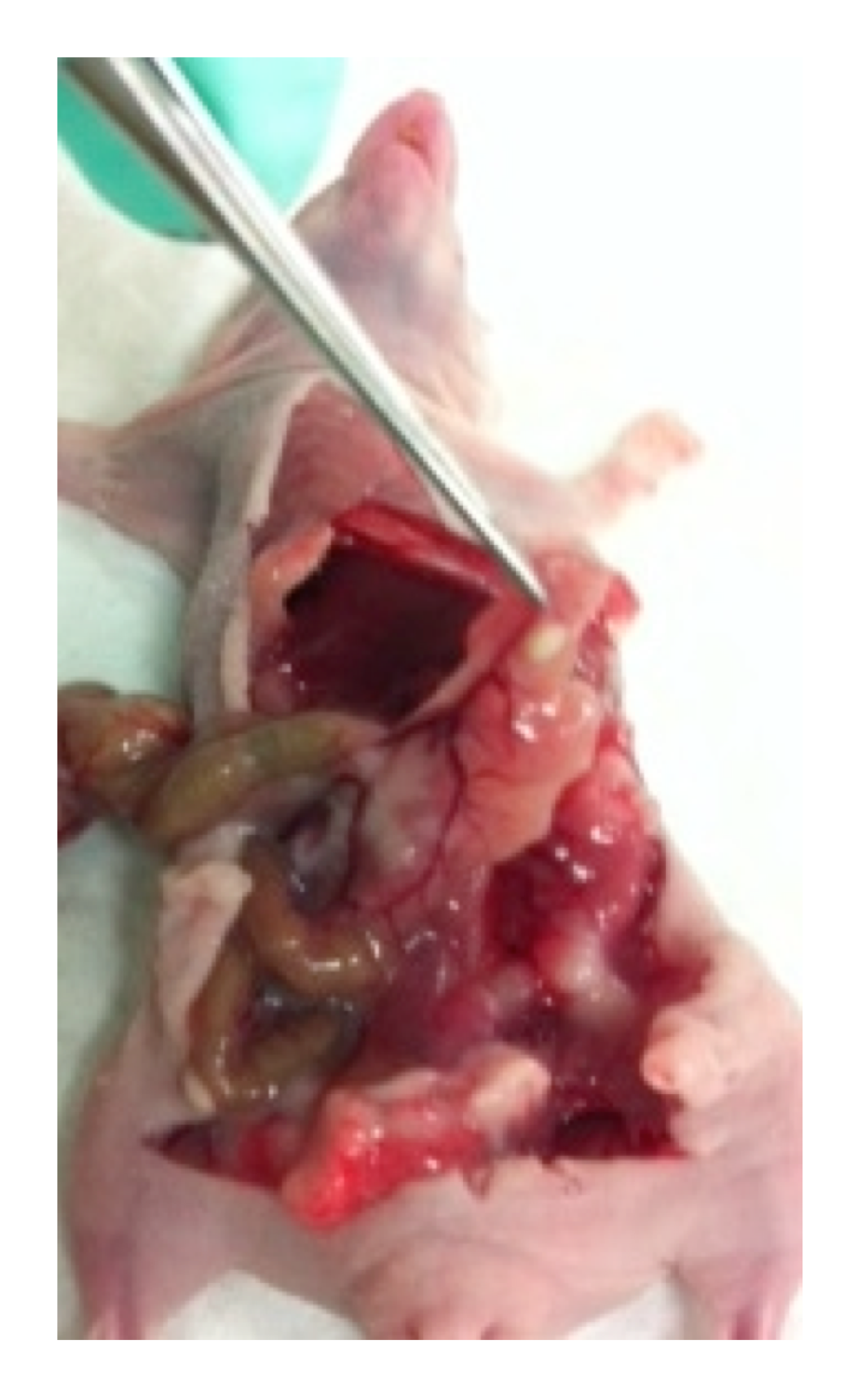

Supplement: Figure S2 — Carcass of ES-2-luc-bearing xenograft model on day 25 post IP inoculation of ES-luc cells (1×10^6^ cells/animal). (TIFF) [file pone.0089968.s002.tiff]
